# A Case Report of a Gluteus Hematoma Detected on ^99m^Tc-MDP Bone Scan

**DOI:** 10.3390/diagnostics12061383

**Published:** 2022-06-03

**Authors:** Miju Cheon, Jang Yoo

**Affiliations:** Department of Nuclear Medicine, Veterans Health Service Medical Center, Seoul 05368, Korea; jang8214.yoo@gmail.com

**Keywords:** hematoma, bone scan, extraosseous radioactivity

## Abstract

This is a case report of a gluteal hematoma detected on ^99m^Tc-methylene diphosphonate (MDP) whole-body bone scan in a 77-year-old male who had experienced a road traffic accident.

A 77-year-old man visited our hospital’s emergency room with severe low back pain. A few hours ago, while towing a cart, he was in a car accident wherein a vehicle collided behind him. The patient had a history of coronary artery disease for which he was taking an anticoagulant. Several tests were performed, including a CT and MRI of the spine. There were multiple transverse processes, and vertebral body fractures in the examination, so conservative management was performed. Additionally, the MRI image of the lumbar spine showed a curvilinear shape lesion with increased signal intensity in the left buttock region (Figure 1). Similar findings to the above were also seen on abdominal CT (Figure 2). Thus, we considered a subcutaneous edema or hematoma for this finding.

Despite conservative management, he complained of severe pain in the left hip. On the fifth day of admission, a whole-body bone scan was performed to determine if there was any other fracture. A technetium-99m methylene diphosphonate (MDP) whole-body bone scan showed an increased uptake noted in the left buttock and left flank and no other site of fracture (Figure 3). The area where the radioactivity was increased matched the location and shape of the lesion where the signal change was seen on the MRI. Furthermore, upon physical examination, we can see a considerable ecchymosis involving the left lateral chest region extending to the left buttock (Figure 4). Mild tracer uptake was also noted in the left facet joint of L4/L5, which is consistent with significant degenerative arthrosis seen on recent lumbar CT images. The osteoid formation and dystrophic calcification are thought to be the mechanisms that mediated a significant number of radiopharmaceuticals in the hematoma on whole-body bone scans [1]. The pathogenesis of the uptake of bone scanning agents in soft tissue is multi-factorial. One of the primary underlying factors is excess calcium in the soft tissue. Mechanisms leading to increased extraosseous ^99m^Tc-MDP uptake include excess calcium in the soft tissue, extracellular fluid expansion, enhanced regional vascularity and permeability, and elevated tissue calcium concentration [2]. In this case report, ^99m^Tc-MDP uptake in gluteal hematoma is thought to be due to excess calcium and enhanced permeability in the soft tissue. Although there is no visible calcification, the accumulation of bone tracer is possible even by microcalcification. Finally, a hematoma was confirmed based on several imaging modalities. Our patient was managed conservatively with analgesic and stopping anticoagulants. The hematoma in the soft tissue of the gluteal region is a rare occurrence and is usually seen in patients taking an oral anticoagulant, having obesity, and facing falls. Contrasted with MRI or CT findings, we could not find any fracture on the bone scan. This false-negative finding for fracture is considered attributable to the fact that for older adults, the result of bone scans may appear normal even ten days after the fracture [3]. It is well established that SPECT (single-photon emission computed tomography) or SPECT/CT (SPECT/computed tomography) has better accuracy than planar scintigraphy. SPECT/CT especially reduces the rate of equivocal lesions compared to planar bone scan due to better anatomic localization of lesions and higher lesion-to-background contrast, with increased diagnostic accuracy over SPECT alone or planar scintigraphy alone [4]. These can contribute to identifying early fractures. If SPECT or SPECT/CT had been performed on this patient, early fracture findings showing only a very slight increase in tracer uptake might have been discovered. Unfortunately, we did not have the equipment in our institution, so SPECT/CT could not be performed on the patient.

This case emphasizes the importance of suspicion of the presence of other lesions in a whole-body bone scan to diagnose a traumatic patient. When reading whole-body bone scans, attention should be paid to the abnormal radioactivity of soft tissues and the skeletal system. This paper is the first report of gluteal hematoma in a whole-body bone scan to the best of our knowledge. There were only a few reports on soft tissue hematoma on other sites, such as brachialis muscle and chest wall hematoma findings on whole-body bone scans [5,6]. A whole-body bone scan may be one of the most valuable diagnostic tools to assess the multiple numbers of traumatic lesions in the whole skeletal system but also traumatic soft tissue lesions such as hematoma.

## Figures and Tables

**Figure 1:**
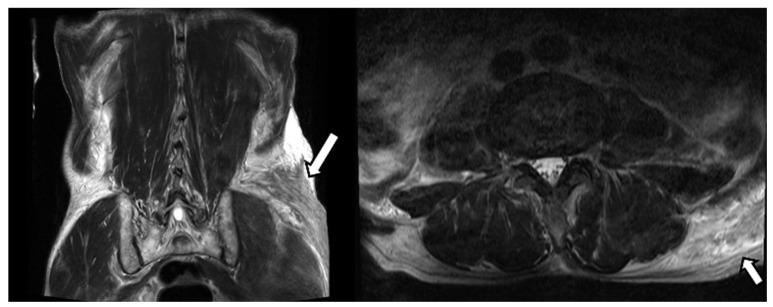
Magnetic resonance imaging (MRI) of the buttock. MRI revealed a well-defined mass in the left buttock. The mass showed a heterogeneously higher signal intensity than the skeletal muscle on T2-weighted images.

**Figure 2:**
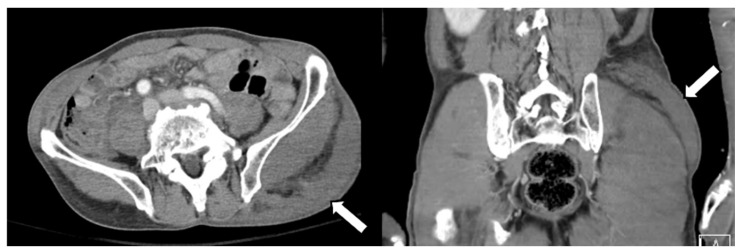
CT scans of the abdomen and pelvis showed an abnormal soft tissue lesion in the left buttock.

**Figure 3:**
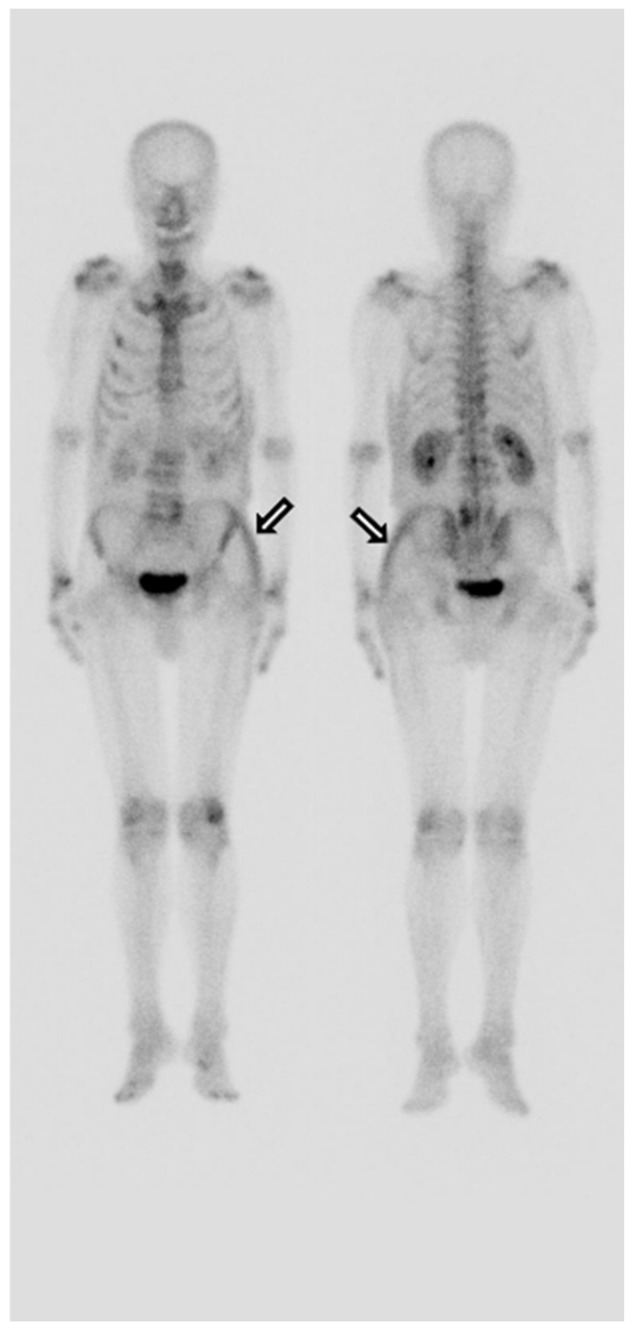
Technetium-99m methylene diphosphonate (MDP) whole-body bone scan shows a curvilinear-shaped hyperactive lesion in the left buttock and left flank and no other fracture site.

**Figure 4:**
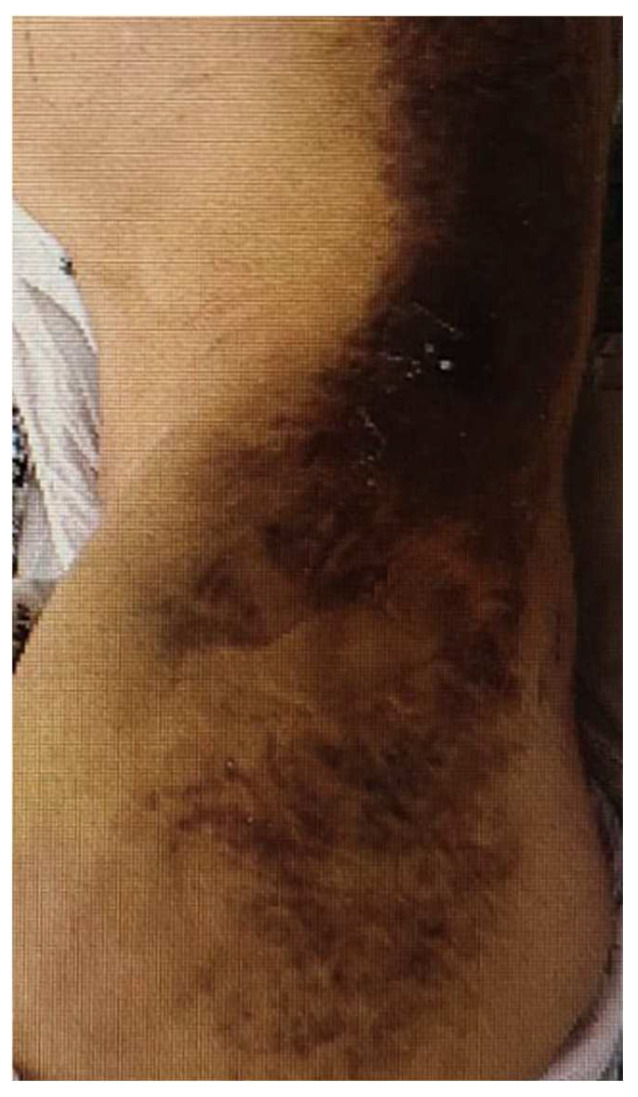
On physical examination, the left flank and left hip showing ecchymosis.

## Data Availability

The data that support the findings of this study are available from the corresponding author, M.C., upon reasonable request.

## References

[1] Wale D.J., Wong K.K., Savas H., Kandathil A., Piert M., Brown R.K. (2015). Extraosseous Findings on Bone Scintigraphy Using Fusion SPECT/CT and Correlative Imaging. AJR Am. J. Roentgenol..

[2] Peller P.J., Ho V.B., Kransdorf M.J. (1993). Extraosseous Tc-99m MDP uptake: A pathophysiologic approach. Radiographics.

[3] Lee K.J., Jung K., Kim J., Kwon J. (2014). Bone scan as a screening test for missed fractures in severely injured patients. Orthop. Traumatol. Surg. Res..

[4] Zhang Y., Shi H., Gu Y., Xiu Y., Li B., Zhu W., Chen S., Yu H. (2011). Differential diagnostic value of single-photon emission computed tomography/spiral computed tomography with Tc-99m-methylene diphosphonate in patients with spinal lesions. Nucl. Med. Commun..

[5] Kamaleshwaran K.K., Mohanan V., Madhavan D., Shinto A.S. (2013). Technetium-99m methylene diphosphonate uptake in the brachialis muscle hematoma in a patient with prostate cancer and coagulation disorder mimicking bone metastasis evaluated by single-photon emission tomography-computed tomography/computed tomography. Indian J. Nucl. Med..

[6] Dhingra J., Baum Y. (2021). Bone Scanning Findings in a Patient with Heat Stroke-Induced Rhabdomyolysis. J. Nucl. Med. Technol..

